# Physical activity intervention for elderly patients with reduced physical performance after acute coronary syndrome (HULK study): rationale and design of a randomized clinical trial

**DOI:** 10.1186/s12872-018-0839-8

**Published:** 2018-05-21

**Authors:** Elisabetta Tonet, Elisa Maietti, Giorgio Chiaranda, Francesco Vitali, Matteo Serenelli, Giulia Bugani, Gianni Mazzoni, Rossella Ruggiero, Jonathan Myers, Giovanni Quinto Villani, Ursula Corvi, Giovanni Pasanisi, Simone Biscaglia, Rita Pavasini, Giulia Ricci Lucchi, Gianluigi Sella, Roberto Ferrari, Stefano Volpato, Gianluca Campo, Giovanni Grazzi

**Affiliations:** 1 Cardiology Unit, Azienda Ospedaliera Universitaria di Ferrara, Ferrara, Cona FE Italy; 2 Department of Medical Science, University of Ferrara, Ferrara, Italy; 3 Department of Public Health, AUSL Piacenza, and Sport Medicine Service, Piacenza, Italy; 4 Center of Biomedical Studies applied to Sport, Public Health Department, Azienda USL di Ferrara, Ferrara, Italy; 5 VA Palo Alto Health Care System, Stanford University School of Medicine, Stanford, CA USA; 6 Cardiology Unit, Ospedale S, Giovanni da Saliceto, Piacenza, Italy; 7 Department of Medicine, Division of Cardiology, “Delta” Hospital AUSL Ferrara, Ferrara, Italy; 8 Division of Cardiology, S. Maria Delle Croci Hospital, Ravenna, Italy; 9 Sport Medicine Center, Ravenna, Italy; 10 Maria Cecilia Hospital, GVM Care and Research, Cotignola, RA Italy

**Keywords:** Acute coronary syndrome, Physical activity, Short physical performance battery, Handgrip

## Abstract

**Background:**

Reduced physical performance and impaired mobility are common in elderly patients after acute coronary syndrome (ACS) and they represent independent risk factors for disability, morbidity, hospital readmission and mortality. Regular physical exercise represents a means for improving functional capacity. Nevertheless, its clinical benefit has been less investigated in elderly patients in the early phase after ACS. The HULK trial aims to investigate the clinical benefit of an early, tailored low-cost physical activity intervention in comparison to standard of care in elderly ACS patients with reduced physical performance.

**Design:**

HULK is an investigator-initiated, prospective multicenter randomized controlled trial (NCT03021044). After successful management of the ACS acute phase and uneventful first 1 month, elderly (≥70 years) patients showing reduced physical performance are randomized (1:1 ratio) to either standard of care or physical activity intervention. Reduced physical performance is defined as a short physical performance battery (SPPB) score of 4–9. The early, tailored, low-cost physical intervention includes 4 sessions of physical activity with a supervisor and an home-based program of physical exercise. The chosen primary endpoint is the 6-month SPPB value. Secondary endpoints briefly include quality of life, on-treatment platelet reactivity, some laboratory data and clinical adverse events. To demonstrate an increase of at least one SPPB point in the experimental arm, a sample size of 226 patients is needed.

**Conclusions:**

The HULK study will test the hypothesis that an early, tailored low-cost physical activity intervention improves physical performance, quality of life, frailty status and outcome in elderly ACS patients with reduced physical performance.

**Trial registration:**

Clinicaltrials.gov, identifier NCT03021044, first posted January, 13th 2017.

**Electronic supplementary material:**

The online version of this article (10.1186/s12872-018-0839-8) contains supplementary material, which is available to authorized users.

## Background

Many evidences suggest that physical activity is associated with benefit in both primary and secondary cardiovascular prevention [1–3]. It is known that the incidence of cardiovascular diseases is age-related and that reduced physical performance and impaired mobility are significantly more frequent in elderly subjects [4]. In these subjects, functional status represents a primary indicator of health status and provides useful prognostic information. Low physical performance is related to higher rates of morbidity, frailty, disability, hospitalization and mortality and, in addition, any hospital admission for acute cardiac events further reduces the overall physical performance in such patients [2, 5]. As a matter of fact, some studies have demonstrated that those who benefit most from physical activity interventions are the elderly, but only few previous studies have enrolled patients aged ≥65 years [6, 7]. This is mainly due to difficulties related to compliance, associated logistic problems and lack of encouragement from physicians [8]. Preserving functional capacity and physical performance in elderly people admitted to the hospital for acute coronary syndrome (ACS) has become more important given the aging of population. ACS remains one of the major causes of mortality and morbidity in Western countries. The mean age of ACS patients has increased progressively in recent years and, although current interventional and medical treatment have significantly improved prognosis, the disability and morbidity burden is still huge*.*

Accordingly, efforts to improve physical performance in elderly patients immediately after hospital discharge for ACS is likely to improve long-term outcomes. It is plausible that an early, tailored and low-cost physical activity intervention in this subset of patients could improve outcomes through the gain of better functional independence. We designed the pHysical activity intervention for elderly with redUced physicaL performance after acute coronary syndrome (HULK) study to address this issue.

## Methods

### Study population

HULK is a prospective, multicenter, interventional, randomized study enrolling patients from three Italian Cardiology units (Azienda Ospedaliera Universitaria di Ferrara, Ferrara; Ospedale Santa Maria delle Croci, Ravenna, Ospedale San Giovanni da Saliceto, Piacenza) and from three outpatient services dedicated to physical activity intervention (Center of Biomedical Studies Applied to Sport, Ferrara; Sport Medicine Center, Ravenna; Sports Medicine Service, Azienda Unità Sanitaria Locale, Piacenza). These are three cardiology units with extensive experience in terms of ACS management and three sports medicine centers with consolidated expertise about physical exercise for cardiopathic patients. A detailed list of inclusion and exclusion criteria is reported in Table 1. Briefly, we include patients aged 70 years or older and admitted to the hospital for ACS with reduced physical performance at time of discharge (T0), confirmed 30-days post-discharge, during the inclusion visit (T1). At the inclusion visit, patients are randomized to the physical activity intervention or to the standard of care groups. Physical performance is assessed with the well validated Short Physical Performance Battery (SPPB) score [9]. Physical performance is considered reduced if SPPB score ranges between 4 and 9 [9–11]. Cardiorespiratory fitness (CRF) is evaluated by the 1-km moderate treadmill walking test (1 k-TWT) [12, 13].

**Table 1:** Inclusion and exclusion criteria

Inclusion criteria	
• Age ≥ 70 years	
• Hospital admission for acute coronary syndrome (ACS) and coronary artery angiography ± percutaneous coronary intervention	ACS is defined in presence of the following criteria (the first mandatory and at least one between criterion 2 and 3): 1. Chest pain suggestive of cardiac origin lasting at least 20 min 2. ECG changes compatibles with signs of myocardial ischemia 3. Detection of rise of cardiac biomarkers
• Informed consent • SPPB score 4–9 at the inclusion visit (T1)	
Exclusion criteria	
• Short portable mental status questionnaire (SPMSQ) < 4	SPMSQ is performed as first test. If it results less than 4 the patient is excluded
• Life expectancy < 12 months	
• The patient is not discharged at home, but he/she is transferred from the cardiology unit to other hospital unit or community structure • Chronic heart failure NYHA III-IV. • Left ventricle ejection fraction < 30%. • Severe aortic or mitral valvulopathy • Multivessel coronary artery disease or left main coronary artery disease candidate to CABG • Planned staged PCI • Impossibility to perform physical activity due to physical impairment	

*ACS* = acute coronary syndrome; *CABG* = coronary artery bypass graft; *PCI* = percutaneous coronary intervention; *SPPB* = short physical performance battery

### Clinical management and study flow

**Fig. 1:**
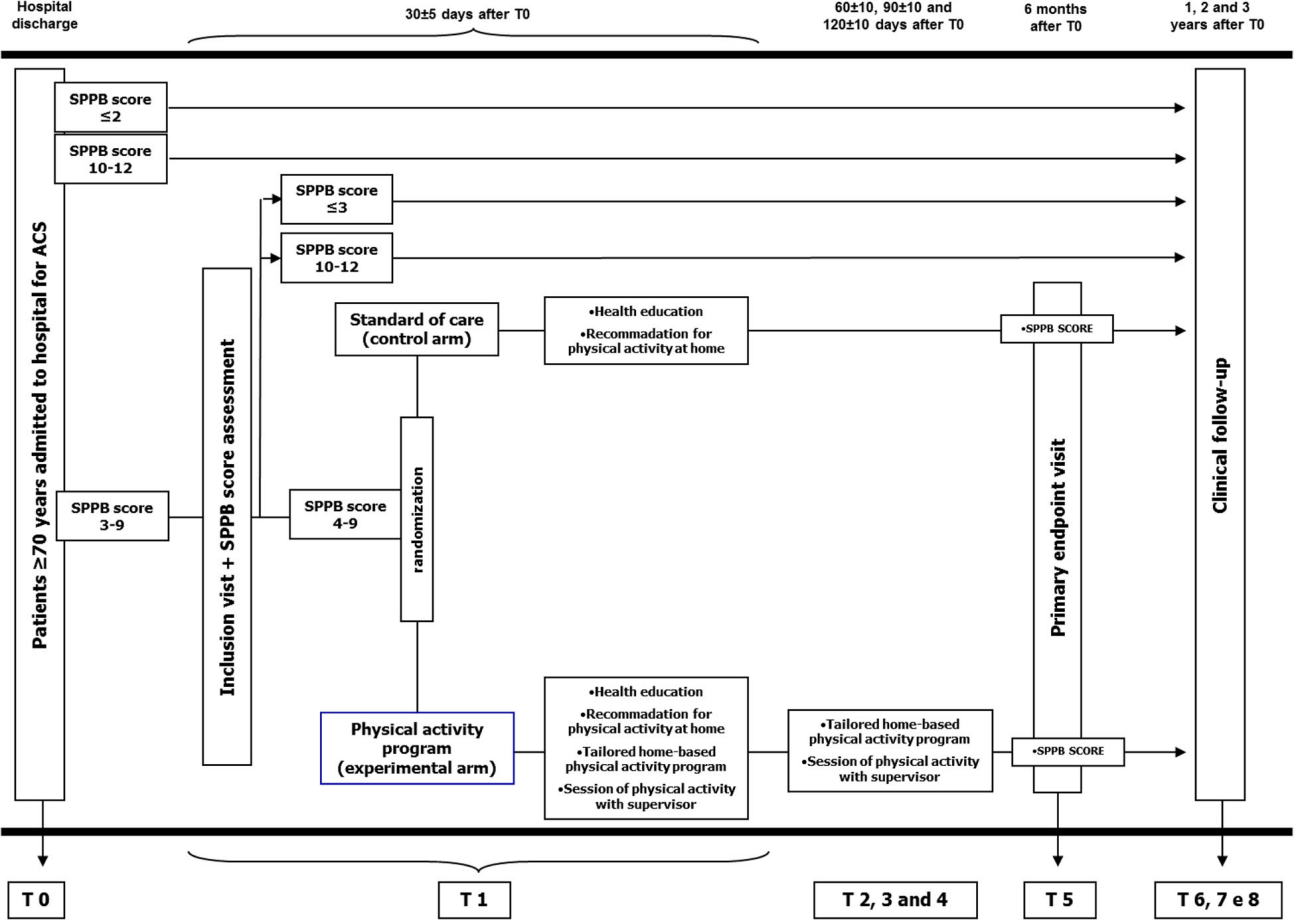
Flow chart of the study. The figure shows the study flow chart for patients admitted to hospital with acute coronary syndrome diagnosis. SPPB: short physical performance battery. ACS: acute coronary syndrome

From hospital admission and ACS diagnosis, all patients are managed according to international guidelines and institutional protocols [14]. Coronary artery angiography (± subsequent coronary revascularization) is performed according to current guidelines [14]. The choice of the management and devices of the coronary revascularization is left to the operator. However, the protocol includes a strong recommendation for complete coronary revascularization with second generation drug eluting stent. Quantitative coronary analysis (QCA), syntax score (SS), residual SS (RSS) and functional SS (FSS) of the index procedure are then also performed. The detailed methodology for calculating QCA and SS are described elsewhere [15–17]. The selection of antiplatelet agent, angiotensin converting enzyme (ACE) inhibitors, beta-blockers and statins is left to the treating physician. The protocol suggests, according to current guidelines, a preferable dual antiplatelet therapy regimen with aspirin and ticagrelor (for at least 12 months), up-titration at the maximal tolerated dose of ACE-inhibitor and lipid lowering treatment to achieve low density lipoprotein (LDL) < 70 mg/dl [14]. In addition, all patients undergo a pre-discharge and 1-year transthoracic echocardiograms to collect full images regarding systolic and diastolic function, valve disease and strain. At the time of the hospital discharge, the investigators propose the enrolment in the study and the informed consent is signed. Blood sampling and a brief comprehensive geriatric assessment including SPPB is administered (Fig. 1). As suggested by guidelines, patients are invited to follow a heart-healthy life style (physical activity, low salt and low fat diet, no smoking) in order to prevent recurrence of cardiovascular events [14]. SPPB score is reassessed at the inclusion visit, 30 ± 5 days after discharge. Only patients confirming a SPPB value between 4 and 9 are randomized. Of note, data of screened but not randomized patients (e.g SPPB value < 4 or > 9 at screening and inclusion visits) are also collected and their clinical follow-up is recorded.

### Randomization

Randomization is performed at the inclusion visit (T1, 30 ± 5 days after hospital discharge), via a dedicated website and stratified according the following three variables: sex, clinical presentation (ST-segment elevation ACS vs. non ST-segment elevation ACS) and SPPB score at the inclusion (4–6 vs. 7–9). A dedicated website assigns a unique treatment code, which dictates the treatment assignment for the subject. Patients are randomized in a 1:1 ratio to standard of care (control group) or to an early, tailored, low-cost physical activity intervention (experimental group). Patients not confirming an SPPB score 4 to 9 thirty days after discharge are excluded (Fig. 1).

### Control group (standard of care)

At the inclusion visit, the investigator stresses again to patients and relatives the major issues related to a heart-healthy life style during a 15-min visit with a study doctor. Specifically, an investigator explains the importance of aerobic physical activity (30–60 min daily, moderate intensity, eg. brisk walking, for at least 3 days/week) to minimize cardiovascular risk [14]. A detailed brochure explaining the benefits of physical exercise is provided to all patients (Additional file 1). To objectively assess leisure time physical activity at home before the follow up visits, each participant is outfitted with a piezoelectric uniaxial accelerometer (MyWellness Key, Technogym, Cesena, Italy) attached at the midline of the right anterior hip; participants are instructed to wear it every time they perform physical activity for a period of 6 months. The MyWellness Key is a simple and valid tool to detect all type of physical activity in free-living settings.

### Interventional group (tailored physical activity intervention)

In addition to standard of care, the experimental group participates in a program of tailored physical activity. Immediately after the inclusion visit, participants are referred to the exercise-based secondary prevention intervention. During the inclusion visit and during the following physical activity sessions each patient performs calisthenics exercises (Additional file 2) and then 1 k-TWT [12]. This protocol has been demonstrated to be a valid and simple tool for physical performance assessment, and it has been shown to predict survival and hospitalization in an outpatient setting [13, 18–21]. A detailed description of the physical activity intervention is illustrated in Table 2. Briefly, during each session subjects are instructed to select a pace that they could maintain for 10 to 20 min at a moderate perceived exercise intensity, (11–13 on the 6–20 Borg scale). Heart rate is monitored continuously and the rate of perceived exertion (RPE) is acquired every 2 min, while test’s difficulty is increasing [12]. After the inclusion visit, activity sessions are scheduled at 60 ± 10, 90 ± 10 and 120 ± 10 days after discharge. All exercise testing and training sessions are performed while continuing the prescribed medications. Participants are instructed to follow the same RPE goals for the home-based exercise program. As the control group, the MyWellness Key accelerometer is distributed to each participant in the interventional group to assess home physical activity, as well as to enhance motivation. The ultimate goal of the intervention is the long-term promotion and maintenance of a physically active lifestyle in order to improve functional ability and physical performance.

**Table 2:** Experimental group: physical activity intervention

Inclusion visit (T1)	Home-based program	Following activity sessions (60 ± 10, 90 ± 10 and 120 ± 10 days after T0)
Pre-test: • measure of blood pressure • positioning RS100 Polar heart rate monitor to constantly evaluate heart rate • Calisthenics exercises	• 30 to 60 min of continuous moderate walking a day, at least 3 to 4 and preferably 7 days a week • Calisthenics exercises^b^	Pre-test: • Measure of blood pressure • Positioning RS100 Polar heart rate monitor to constantly evaluate heart rate. • Evaluation of data recorded by accelerometer. • Calisthenics exercises^b^
Start: walking on the level at 2.0 km/h		Start: walking at an updated intensity estabilished according to reached results in the previous activity session
Every 30 s: increases of 0.3 km/h up to reach a walking speed corresponding to a perceived exertion of 11–13 on the Borg scale for 1 km^a^.		Every 30 s: increases of 0.3 km/h up to reach a walking speed corresponding to a perceived exertion of 11–13 on the Borg scale for 1 km^a^.
Post-test: • Measure of blood pressure. • Counselling on physical activity and daily activities, such as gardening, or household work. • Distribution of home accelerometer		Post-test: • Measure of blood pressure • Counselling on physical activity and daily activities, such as gardening, or household work. • Distribution of home accelerometer

^a^Subjects walking at a perceived moderate speed < 3.0 km/h will perform the test over the distance of 500-m. At the end of the test the averaged walking speed will be calculated

^b^detailed description in the Additional file 2

### Study visits and follow-up

After the inclusion visit (T1, 30 ± 5 days after hospital discharge), patients undergo study visits at 6, 12, 24 and 36 months after discharge. During the study visits any information regarding clinical status, outcome and adverse events is collected. Compliance to medical treatment is assessed by interview.

### Primary endpoint

Primary endpoint is the SPPB score at the 6-month study visit. The SPPB is a short battery of tests for lower limb function [9]. Briefly, the SPPB is composed of three tests: standing balance, usual walking speed and chair sit-to-stand. The standing balance test consists in the ability to maintain the standing position for 10 s with three different foot position: parallel, semi-tandem and tandem. Walking speed evaluates the time needed to progress for 4 linear meters with patient’s usual speed, assigning a different score according to speed. Finally, chair sit-to-stand assesses the ability to stand from a chair 5 consecutive times without using arms. The SPPB score ranges from 0 (worst performance) to 12 (best performance). It has been known that the test has a strong and independent ability to predict mortality, morbidity and hospitalization [10, 11]. Many previous studies investigating the benefit of physical activity intervention employed the SPPB score as an endpoint [22–24]. The test is performed by trained study investigators. To ensure that patients are able to execute commands, a rapid evaluation of cognitive impairment is performed using the Short Portable Mental Status Questionnaire (SPMSQ) [25]. Of note, the staff performing SPPB score and other tests at the 6-month visit is different from that of the inclusion visit and it is blinded regarding treatment (experimental vs. standard care).

### Secondary endpoints

Secondary endpoints include several different tests and the collection of clinical adverse events across the entire study time.

#### Handgrip strength (HGT)

The HGT involves the use of a dynamometer to measure the force of contraction of the flexor muscles of the fingers, of muscles of the wrist and forearm. HGT is associated with nutrition status of the subject, and with functional recovery post-surgery [26]. Each patient is asked to squeeze the dynamometer three different times and the highest of the three is used to reflect HGT. The measurement is performed with the patient seated, with the dominant hand and the elbow flexed 90° [26]. All study staff is educated regarding correct HGT screening. In all centers a Jamar hydraulic hand dynamometer (Patterson Medical, Warrenville, Il, USA) is used. The results are expressed in Kilograms force.

#### EuroQol-5D, Quality of life questionnaire

The EuroQol-5D is a simple instrument to evaluate health status and quality of life [27, 28]. It has been previously validated in patients with cardiovascular disorders, including participants in rehabilitation programs [29–32]. The EQ-5D instrument measures health status in 5 dimensions: mobility, self-care, usual activities, pain/discomfort, and anxiety/depression. Each dimension is rated according to the following levels: i) no problems; ii) some problems; iii) extreme problems. The questionnaire employs a Visual Analogue Scale (VAS) to quantify perceived health. There is also an algorithm to collect all data in a single score [29]. It is administered during the inclusion visit as a basal value and at 6, 12, 24, 36 months.

#### Advanced Activity Daily Living (aADL)

Advanced activities of daily living are social and physical activities that are reduced with aging. These reductions are considered an index of functional decline that appears before ADL and IADL loss. These activities include walking, gardening, hobbies, social and group activities. Besides, the aADL evaluation is related to functional capacity and long-term prognosis [33]. The aADL evaluation is characterized by two simple questionnaires created by Rosow et al. (evaluation of social and group activities) and Reuben et alt. (physical activity frequency) [33, 34]. Data about aADL are collected at hospital admission in clinical medical notes and then during follow up visits at 6, 12, 24 and 36 months.

#### 7-Day Physical Activity Recall Questionnaire (PAR)

This questionnaire estimates the individual’s time engaged in physical activity including aerobic, strength, and flexibility activities for the 7 days prior to the interview. It quantifies duration and intensity of physical activities. Only physical activities of moderate intensity and greater are counted. From hours spent in moderate, hard, and very hard intensity physical activities, total kilocalories/day can be estimated [35]. This questionnaire is administered in both groups during the inclusion visit to assess baseline levels of physical activity. These data are also used as a basal value in the interventional group to decide the starting physical activity level during the first session and then at 6, 12, 24 and 36 months, making up a secondary endpoint.

#### Adverse events

All adverse events such as all-cause death, cardiovascular death, all-cause re-hospitalization and cardiovascular re-hospitalizations are recorded across the entire follow-up period. A detailed list of all clinical adverse events collected is reported in Table 3. Of note, all adverse events will be assessed by an independent, blinded adjudication committee, according to current consensus documents [36, 37].

**Table 3:** Study endpoints

	6 month after T0	1, 2 and 3 years after T0
Primary endpoint		
• SPPB score	**X**	
Secondary Endpoints		
• SPPB score		**X**
• Handgrip test		**X**
• All-cause mortality		**X**
• Cardiovascular mortality		**X**
• All-cause re-hospitalization		**X**
• Cardiovascular re-hospitalization		**X**
• aADL score	**X**	**X**
• PAR Questionnaire	**X**	**X**
• LDL- cholesterol level		**X**
• EQ-5D score	**X**	**X**
• Glycated Haemoglobin (HbA1C) level	**X**	**X**
• On treatment platelet reactivity	**X**	**X**
• Nuisance bleedings	**X**	**X**

*aADL* = advanced activity daily living; *EQ-5D* = EuroQol 5D; PAR = 7-day physical activity recall; *SPPB* = short physical performance battery

#### Blood sampling

At hospital discharge and during all follow up visits, venous blood sample is collected in all patients from an antecubital vein using a 21-gauge needle. The first 2–4 mL of blood are discarded. The remaining is used to obtain DNA, plasma, and serum samples which are stored. In addition, platelet function assays are performed at all time-points. On-treatment platelet reactivity is evaluated with light transmission aggregometry after stimulus with adenosine diphosphate and arachidonic acid [38–42]. Laboratory data (i.e. LDL-cholesterol levels and glycated haemoglobin value) are also collected during all follow-up visits.

### Data collection and management

In each Cardiology unit study investigators collect all data regarding baseline characteristics, laboratory data, medical/interventional treatments and follow-up. All data are entered into a web-based electronic case report form. Data management and coordination are performed by Centro di Epidemiologia Clinica della Scuola di Medicina at the University of Ferrara (Ferrara, Italy) (see study organization in the Additional file 3). The study is initiated and supported by the Cardiovascular Center of the University of Ferrara. All data are transmitted in anonymous form to the coordinator center. A reference number is assigned to each patient enrolled.

### Statistical analysis

Continuous data is tested for normal distribution with the Kolmogorov-Smirnov test. Normally distributed values are presented as mean ± SD and are compared by t test and 2-way ANOVA; otherwise as median value [interquartile range], and the Mann-Whitney U and Kruskal-Wallis tests are used. Categorical variables are summarized in terms of number and percentages and are compared by using Pearson’s chi-squared or two-sided Fisher’s exact tests. Survival curves are generated by the Kaplan- Meier method, and differences are evaluated using the log-rank test. Cox proportional hazard regression models are run to identify independent predictors of adverse events. All tests are 2-sided and the statistical significance is defined as *p* < 0.05. All analyses are performed with *Stata 13* and with R 3.1.2 by the staff (EM) of the Center for Clinical Epidemiology of the School of Medicine at the University of Ferrara (Ferrara, Italy).

### Sample size calculation

Taking into account previous studies and preliminary findings from the FRASER program [11, 16], we expect to find in the experimental arm an increase of at least one point in the 6-month SPPB score, as compared to standard of care. Assuming a standard deviation of the SPPB score of 2.5, in order to obtain a statistical power ≥ 80% (alpha 5%), we would require an overall sample size of 226 patients.

### Trial status

This study is led in accordance with the amended Declaration of Helsinki. The study protocol has been approved by the hospitals’ ethics committees prior to the study beginning (November 2016, study ID 161098). An approved written informed consent is obtained from all patients at the time of enrolment. The protocol is registered on ClinicalTrial.gov with the number: NCT03021044 (January, 13th 2017). The study started in January 2017 (January, 16th: first patient enrolled) and is currently ongoing. The achievement of the sample size target is planned for March 2018 and the initial results will be available in October 2018.

## Discussion

The HULK study is designed to evaluate the benefits of a tailored physical activity intervention on functional capacity and physical performance in elderly patients after ACS. The major strengths of our study are: i) a population reflecting the real-life scenario; ii) the inclusion of high risk patients enrolled after an acute event; iii) the evaluation of an early tailored low-cost physical activity intervention; iv) the multi-parametric assessment of physical performance and functional capacity.

In a cardiovascular setting, elderly patients have more complications, more impaired physical function and higher risk than younger patients; hence interventions including health education, clinical follow up, prevention of re-hospitalization and prescription of tailored exercise are more necessary. These particular patients are usually understudied and underrepresented in large randomized trials [7]. In fact, the few prospective studies that have evaluated the effects of physical training in geriatric patients have shown significant improvements in functional ability, which is highly relevant for the prevention of disability, and improving of the quality of life [6, 7] [43, 44]. The U.S. Medicare population registry shows that in elderly patients physical training interventions have positive prognostic effects, with a “dose-response” relationship, in which the dose corresponds to the number of sessions of the program performed by patients [2, 5]. Data from the LIFE study provided evidence that structured physical activity interventions improve the SPPB score in frail elderly subjects, suggesting that physical activity interventions might have the potential to reduce the occurrence of disability [23, 24, 44]. SPPB is a highly sensitive indicator of global health status reflecting several underlying physiological impairments [12]. As shown by a recent meta-analysis, SPPB is also predictive of all-cause mortality in a dose-response manner [45]. Regarding patients with cardiovascular disease, Weibel et al. demonstrated that education and counseling for patients with ACS should occur early after discharge with the aim of promoting compliance to physical activity programs [46]. Revees et al. in a recent pilot study observed that in elderly patients admitted for acute heart failure participating in a physical activity program, the change of SPPB score was strongly and inversely related to all-cause re-hospitalizations [22]. The HULK study summarizes and extends all these experiences. The attention is focused on elderly frail patients. The physical activity intervention is begun early after hospital discharge and it provides outpatient tailored physical activity sessions without prolonging hospitalization. The direct comparison of the HULK program with standard cardiac rehabilitation programs was beyond the aim of this trial. The HULK study reflects the real-life scenario in which usually elderly coronary patents are not referred to cardiac rehabilitation programs or show a very low compliance to this type of secondary prevention. For this reason, the control arm did not include any kind of supervised physical intervention. With this background the HULK study could offer a feasible alternative for elderly patients’ improvement in terms of physical performance and functional capacity. Taking into account these concepts, an important role is played by the home-based component that allows patients to make tailored physical activity a habit and maintain their improvement. So the HULK program could help to overcome the main limitations of the current cardiac rehabilitation, such as the high number of sessions, high costs, low patients’ compliance and the lacking long-term maintenance of an active lifestyle.

## Conclusions

The HULK study aims to investigate whether an early, tailored, low-cost physical activity intervention improves physical performance, quality of life and delays the onset of disability in elderly ACS patients with reduced functional capacity.

## Additional files

Additional file 1: Brochure for patients at hospital discharge. (DOC 499 kb)

Additional file 2: Calisthenics exercises. (DOC 1084 kb)

Additional file 3: Study organization and full list of investigators. (DOC 62 kb)

## Abbreviations

1 k-TWT: 1 km-Treadmill Walking Test
aADL: Advanced Activity Daily Living
ACE: Angiotensin Converting Enzyme
ACS: Acute Coronary Syndrome
CRF: Cardiorespiratory Fitness
EQ-5D: EuroQol 5D
FSS: Functional Syntax Score
HGT: Handgrip Test
LDL: Low Density Lipoprotein
PAR: 7-day Physical Activity Recall Questionnaire
QCA: Quantitative Coronary Analysis
RSS: Residual Syntax Score
SPMSQ: Short Portable Mental Status Questionnaire
SPPB: Short Physical Performance Battery
SS: Syntax Score
VAS: Visual Analogue Scale
