# Supplementary material for: Physical activity intervention for elderly patients with reduced physical performance after acute coronary syndrome (HULK study): rationale and design of a randomized clinical trial
Source: BMC Cardiovasc Disord. 2018 May 21;18:98. doi: 10.1186/s12872-018-0839-8 (PMC5963011; doi:10.1186/s12872-018-0839-8)
Supplement: Supplementary file 2 — Calisthenics exercises. (DOC 1084 kb) [file 12872_2018_839_MOESM2_ESM.doc]

**Calisthenic exercises**

*Seated free body exercises (6 exercises repeated 2 times)*

***Type 1***


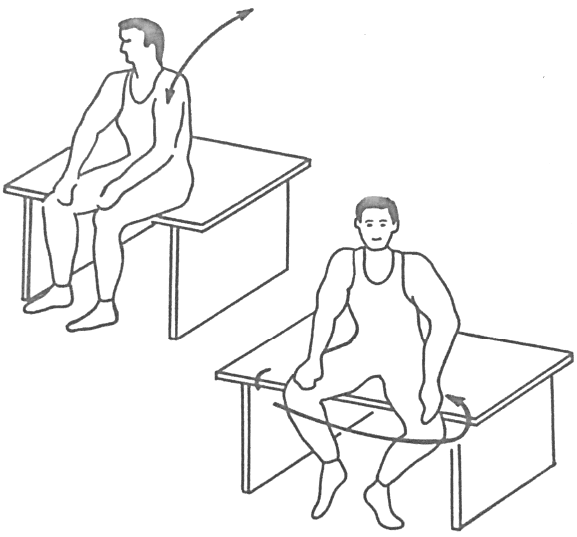


1. Breathe out and close your knees
2. Breathe in and spread your knees

***Type 2***


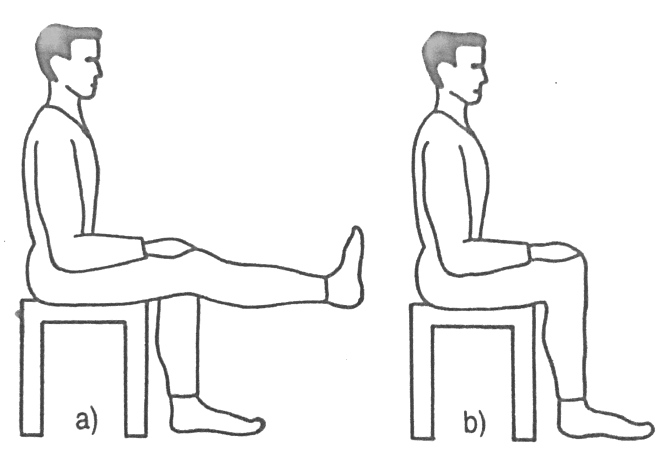


1. Breathe in and stretch the right leg
2. Breathe out and return to the starting position
3. Breathe in and stretch the left leg
4. Breathe out and return to the starting position

***Type 3***


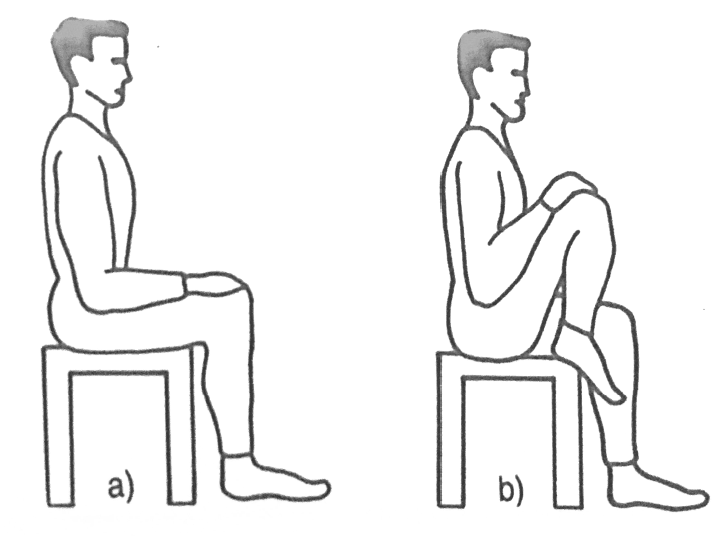


1. Breathe in
2. Breathe out and bend your right leg
3. Breathe in
4. Breathe out and bend your left leg

***Type 4***


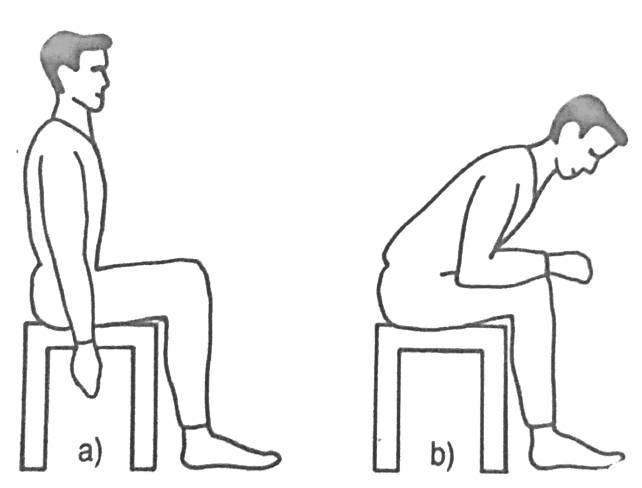


1. Breathe in with straight back
2. Breathe out and bend forward your back

*Standing free body exercises (6 exercises repeated 2 times)*

***Type 1***


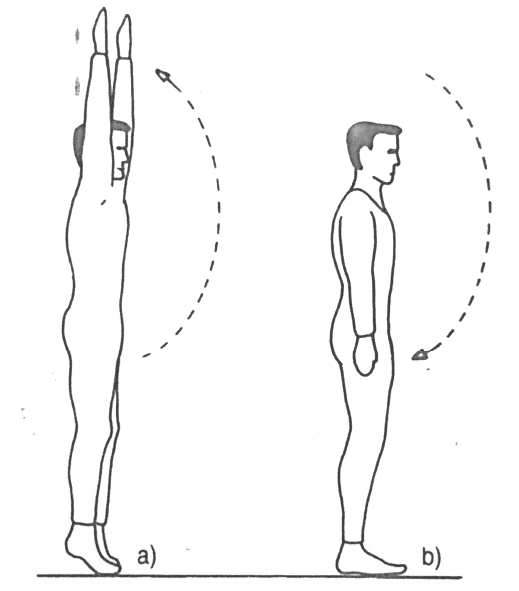


1. Breathe in bringing forward your arms
2. Breathe out bringing your arms down

***Type 2***


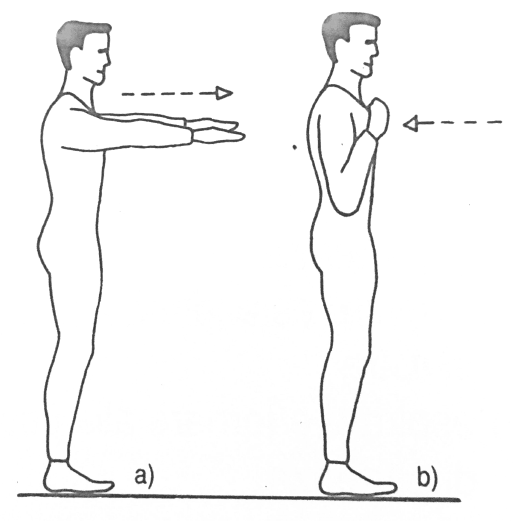


1. Breathe in and bring your arms stretched forward
2. Breathe out bringing your arms back to the chest

***Type 3***


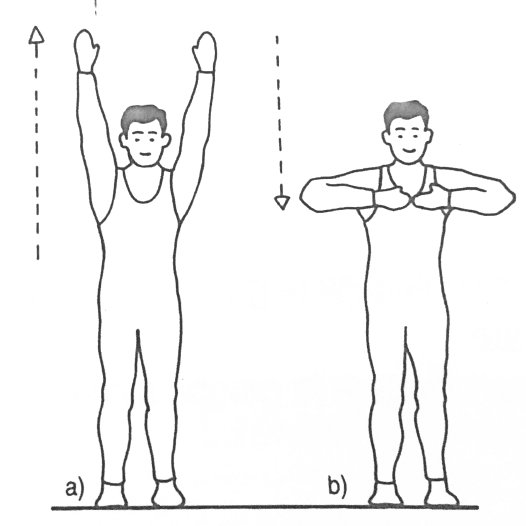


1. Breathe in bringing your arms stretched to the top
2. Breathe out bringing your arms at shoulder height

***Type 4***


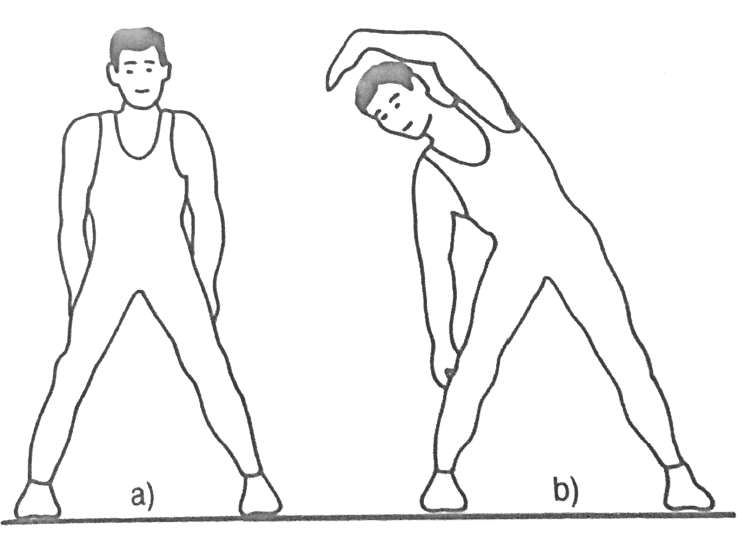


1. Breathe in spreading your legs
2. Breathe out and bend sideways your bust

***Type 5***


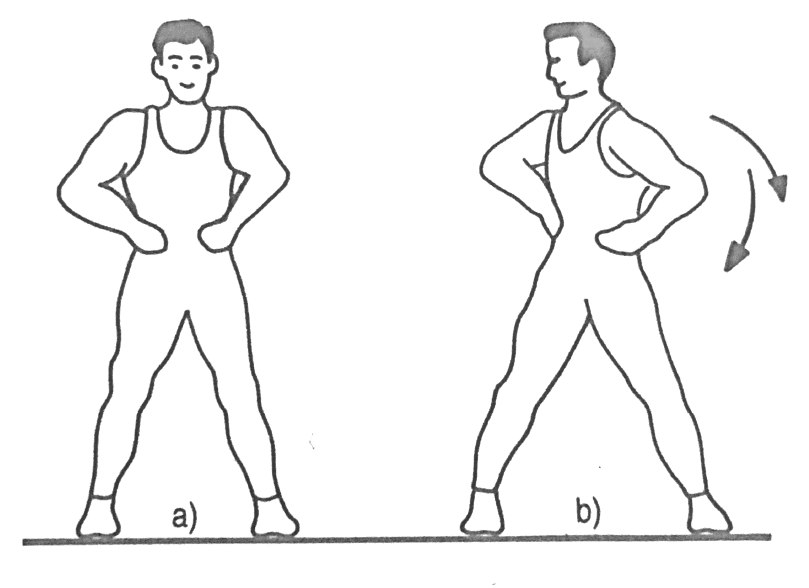


1. Breathe in spreading your legs
2. Breathe out during torsion of your bust

***Type 6***


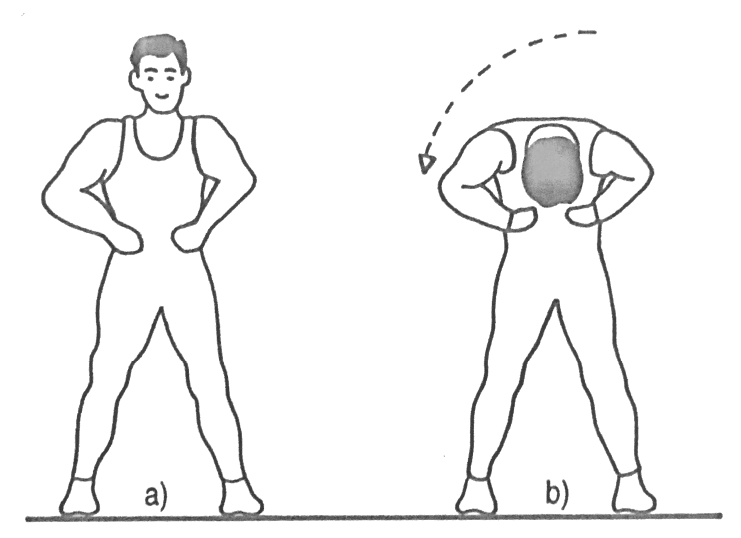


1. Breathe in
2. Breathe out and bend forward your bust

***Type 7***


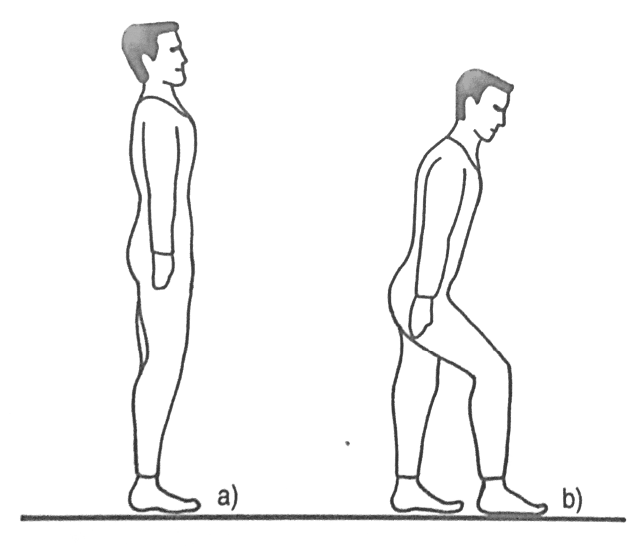


1. Breathe in
2. Breathe out stepping forward

***Type 8***


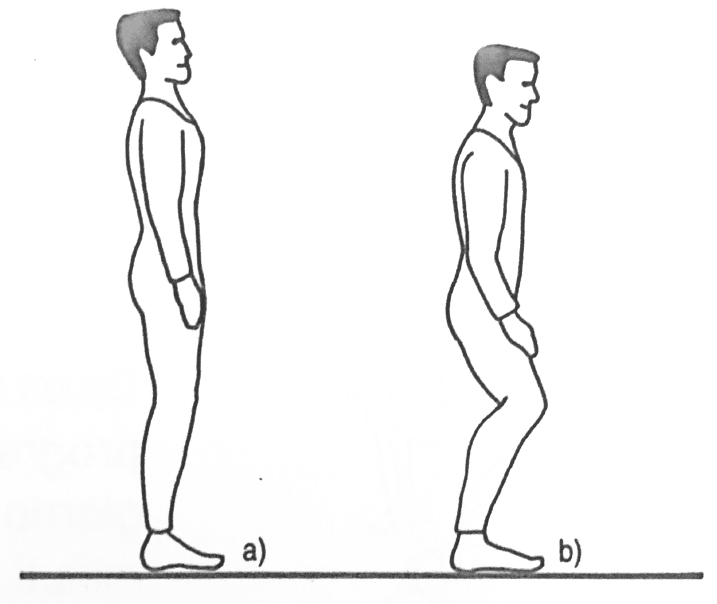


1. Breathe in
2. Breathe out and bend your knees
