# Supplementary material for: Physical activity intervention for elderly patients with reduced physical performance after acute coronary syndrome (HULK study): rationale and design of a randomized clinical trial
Source: BMC Cardiovasc Disord. 2018 May 21;18:98. doi: 10.1186/s12872-018-0839-8 (PMC5963011; doi:10.1186/s12872-018-0839-8)
Supplement: Supplementary file 3 — Study organization and full list of investigators. (DOC 62 kb) [file 12872_2018_839_MOESM3_ESM.doc]

*Steering committee:*

Gianluca Campo, MD, Cardiovascular Institute, Azienda Ospedaliero-Universitaria di Ferrara, Italy; Stefano Volpato, MD, Department of Medical Sciences, University of Ferrara, Italy; Giovanni Grazzi, MD, Center of Biomedical Studies Applied to Sport, University of Ferrara, Italy; Giorgio Chiaranda, MD, Sports Medicine Service Piacenza, Public Health Department, AUSL Piacenza, Italy.

*Clinical Events adjudication:*

Rita Pavasini, MD, Cardiovascular Institute, Azienda Ospedaliero-Universitaria di Ferrara, Italy;

Annamaria Del Franco, MD, Cardiovascular Institute, Azienda Ospedaliero-Universitaria di Ferrara, Italy.

*Data Management and Monitoring:*

Veronica Lodolini, BSc, Cardiovascular Institute, Azienda Ospedaliero-Universitaria di Ferrara, Italy; Elisa Mosele, PhD, Cardiovascular Institute, Azienda Ospedaliero-Universitaria di Ferrara, Italy; Elisa Maietti, PhD, Centro di Epidemiologia Clinica della Scuola di Medicina, University of Ferrara, Italy.

*Statistical committee:*

Stefano Volpato, MD, Department of Medical Sciences, University of Ferrara, Italy; Elisa Maietti, PhD, Department of Medical Sciences, University of Ferrara, Italy.

*Core transthoracic ecocardiography Laboratory:*

Jlenia Marchesini, MD, Cardiovascular Institute, Azienda Ospedaliero-Universitaria di Ferrara, Italy; Paolo Cimaglia, MD, Cardiovascular Institute, Azienda Ospedaliero-Universitaria di Ferrara, Italy.

*Core coronary artery angiography Laboratory:*

Simone Biscaglia, MD, Cardiovascular Institute, Azienda Ospedaliero-Universitaria di Ferrara, Italy; Francesco Gallo, MD, Cardiovascular Institute, Azienda Ospedaliero-Universitaria di Ferrara, Italy.

*Full list of Investigators:*

Cardiovascular Institute, Azienda Ospedaliero-Universitaria di Ferrara:

- Elisabetta Tonet, Matteo Serenelli, Giulia Bugani, Francesco Vitali, Rossella Ruggiero

Center of Biomedical Studies Applied to Sport, University of Ferrara:

- Giovanni Grazzi, Gianni Mazzoni

Cardiology Unit, Ospedale San Giovanni da Saliceto, Piacenza:

- Giovanni Quinto Villani, Massimo Piepoli, Maria Sole Pisati, Deborah Bertoncelli, Paola Pontremoli, Ursula Corvi, Maria Tansini, Gianluca Lisè, Tiziana Leonarda Auletta

Sports Medicine Service, Azienda Unità Sanitaria Locale, Piacenza:

- Giorgio Chiaranda, Fiumara Graziella, Badagni Cinzia, Beato Rossella, Cordani Silvia, Favari Tiziana, Ferri Sara, Lommi Federica, Scolari Lara

Cardiology Unit, Ospedale S. Maria delle Croci, Ravenna, Italy

- Giulia Ricci Lucchi, Giancarlo Piovaccari

Sport Medicine Center Ravenna:

Gianluigi Sella
