# Supplementary material for: Physical activity intervention for elderly patients with reduced physical performance after acute coronary syndrome (HULK study): rationale and design of a randomized clinical trial
Source: BMC Cardiovasc Disord. 2018 May 21;18:98. doi: 10.1186/s12872-018-0839-8 (PMC5963011; doi:10.1186/s12872-018-0839-8)
Supplement: Supplementary file 3 [file 12872_2018_839_MOESM1_ESM.doc]

Proper nutrition

Enjoy every day a wide variety of nutrients between the foods listed below:

• Vegetables of different types and colors, and legumes/beans

• Fruit

• Grain (cereal) foods, mostly wholegrain and/or high cereal fibre varieties, such as breads, cereals, rice, pasta, noodles, polenta, couscous, oats, quinoa and barley

• Lean meats and poultry, fish, eggs, tofu, nuts and seeds, and legumes/beans

• Milk, yoghurt, cheese with reduced fat

And drink water.

Limit intake of foods containing saturated fat, added salt, added sugars and alcohol such as biscuits, cakes, pastries, pies, processed meats, commercial burgers, pizza, fried foods, potato chips, crisps and other savory snacks.

Healthy Eating!

SERVIZIO SANITARIO REGIONALE
EMILIA-ROMAGNA
Azienda Ospedaliero - Universitaria di Ferrara

università di ferrara
DA SEICENTO ANNI GUARDIAMO AVANTI.

And now that I come home?

Practical indications for patients discharged after acute coronary syndrome

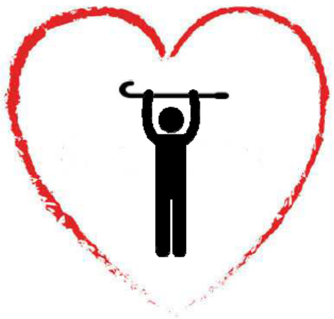

Cardiology Unit, Azienda Ospedaliera Universitaria di Ferrara

If you are reading this brochure it means that you have been hospitalized for acute coronary syndrome. This has happened because in the vessels that carry blood to your heart a fat plaque has broken, obstructing the vessel. Even if that plaque has been settled, you remain at risk of developing other plaques, which may break, causing serious consequences. For this reason, you will need to accurately assume the prescribed medications and perform periodic visits. But it's not over here! To fight fat plaques drugs are not enough, you need to have an healthy lifestyle, based on proper nutrition and physical activity.

First of all, there are some tips on resuming daily activities after hospitalization:

- After hospital discharge, you should stay at home in the first 2-3 days, then you can go out for a short walk, beginning with 15-30 minutes a day and gradually increasing the duration of the exercise the following days
- Cigarette smoking is not recommended in the most absolute way
- Sexual activity can be started again a few days after discharge
- 4 weeks after discharge you can start again your job, if it isn’t too physically tiring

- Car driving can be resumed 3-4 weeks after the heart attack, without engaging in very long or challenging travel
- You can go on holiday, stopping for some time in case of a long journey, carrying medication and clinical documentation, avoiding altitudes above 1500m and scuba diving, avoiding the plane if you are afraid of flying.

Stop Smoking!

Physical activity

1) Make physical activity a habit
2) Make a graded physical activity plan in order to avoid fatigue and sorrow
3) Vary activities by changing the type of performance or the way to do it
4) Alternate physical activity with adequate rest breaks, needed to regain strength and renew motivation and will to stay active.

You should do at least 30 minutes of physical activity on 5 days of the week

Little suggestions:
-For short trips, walk or cycle and leave the car at home.
-For longer trips, walk or cycle part of the way.
-Use the stairs instead of the lift.
-Get off the bus one stop earlier and walk the rest of the way.
-Park further away from your destination and walk.
-Catch up with friends for a walk, instead of sitting to chat.

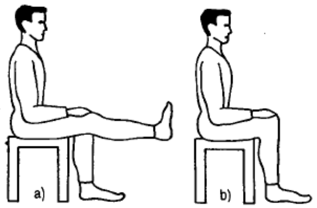

a) Breathe in and stretch the right leg
b) Breathe out and return to the starting position
a) Breathe in and stretch the left leg

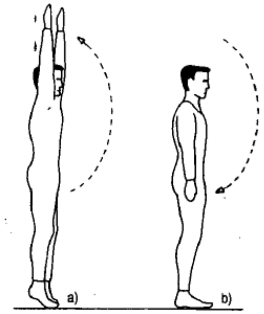

a) Breathe in bringing forward your arms
b) Breathe out bringing your arms down

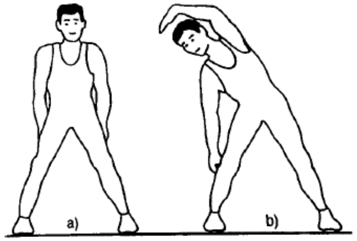

a) Breathe in
b) Breathe out and bend sideways your bust

Moving more and sitting less!
